# Advances in Surface-Enhanced and Tip-Enhanced Raman Spectroscopy, Mapping and Methods Combined with Raman Spectroscopy for the Characterization of Perspective Carbon Nanomaterials

**DOI:** 10.3390/nano13172495

**Published:** 2023-09-04

**Authors:** Marianna V. Kharlamova

**Affiliations:** Faculty of Physics, University of Vienna, Boltzmanngasse 5, A-1090 Vienna, Austria; mv.kharlamova@gmail.com

Surface-enhanced Raman spectroscopy (SERS) is based on the effect of the plasmonic enhancement of intensity of the Raman scattering of molecules in cases when they are adsorbed on a substrate [1,2,3]. SERS is a quick and highly sensitive method [4,5,6,7]. As substrates, graphenes with Au and Ag nanoparticles and graphene oxides with Au and Ag as well as reduced graphene oxides with Au, Ag, and Cu are used. Figure 1 shows the increase in the Raman peak intensities of rhodamine R6G molecules were adsorbed from 10^−6^ M solution on the silvered porous silicon free of graphene and were covered with graphene in the light (the so-called light spot) and in the dark (the so-called dark spot) [8]. Also, metallic (Ag, Au, Cu) nanostructures covered with graphenes as well as nanostructures covered with graphene oxides can be used. SERS has also been also used for the characterization of carbon nanotubes [2,3].

Tip-enhanced Raman spectroscopy (TERS) is based on the effect of surface plasmon enhanced Raman scattering; however, the precisely controlled atomic force microscopy (AFM) tip covered with Au or Ag is employed instead of a substrate with metallic nanoparticles [9,10]. The method is used for the mapping and/or location-specific investigations of wrapped double-layered graphene; the number of graphene layers; impurities on the surface of graphene; defects and borders of graphene layers; mechanical tensions; and graphene doping. The method is also used for the investigation of carbon nanotubes [9,11]. Figure 2 shows the increased Raman peak intensities of multi-walled carbon nanotubes on an Au substrate observed with the TERS tip in close proximity (1–2 nm) to the sample surface and acquired in the AFM mode [11].

Raman mapping allows us to obtain maps with different fitted/extracted peak parameters, such as intensity, positions, full widths at half maximum, and intensity ratios [12].

Raman spectroscopy can be combined with other methods [13,14,15]. In Refs. [16,17], the characterization of effects of the irradiation of graphene via focused ion beam on the structure was investigated using Raman spectroscopy combined with AFM and scanning electron microscopy. In Ref. [18], the diameter distribution of single-walled carbon nanotubes (SWCNTs) was analyzed using Raman spectroscopy combined with transmission electron microscopy.

This Special Issue entitled “Advances in Spectroscopy of Carbon Nanomaterials: Methods and Applications” focuses on the application of spectroscopy for carbon nanomaterials. This Special Issue covers recent progress in the methods and applications of spectroscopy in the investigation of carbon nanotubes, graphene, graphene nanoribbons, 2D heterostructures, fullerenes, nanodiamonds, and other novel nanostructures.

In review paper [19], the authors discuss the applications of spectroscopy for the investigation of carbon materials for electrochemical applications. They discuss electrochemical doping. The spectroscopy experiments in different electrolyte solutions are highlighted. The chemical functionalization of carbon nanotubes for applications is presented. Applications of carbon material in batteries and supercapacitors are considered.

I invite interested authors to submit their best works to the Special Issue entitled “Advances in Spectroscopy of Carbon Nanomaterials: Methods and Applications”.

## Figures and Tables

**Figure 1:**
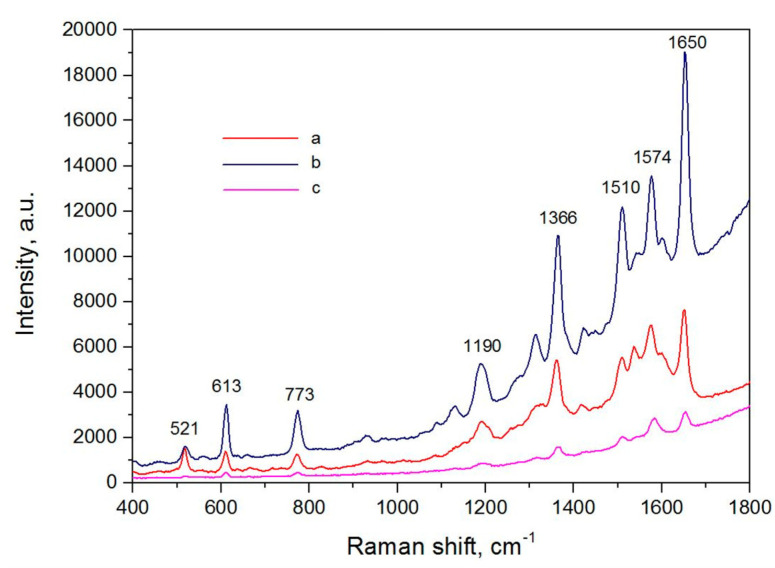
SERS spectra of rhodamine R6G molecules adsorbed from 10^−6^ M solution on the silvered porous silicon: (**a**) free of graphene and covered with graphene in the light spot (**b**) and in the dark spot (**c**). Copyright 2019 by the authors. Licensee MDPI, Basel, Switzerland. This article is an open access article distributed under the terms and conditions of the Creative Commons Attribution (CC BY) license [8].

**Figure 2:**
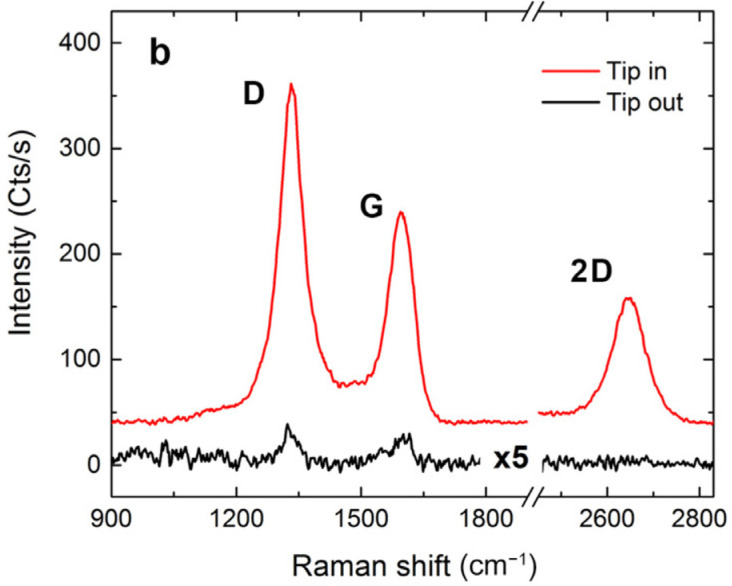
Raman signal of multi-walled carbon nanotubes on the Au substrate observed with or without the TERS tip in close proximity (1−2 nm) to the sample surface and acquired in the AFM mode. Copyright 2022 by the authors. Licensee MDPI, Basel, Switzerland. This article is an open access article distributed under the terms and conditions of the Creative Commons Attribution (CC BY) license [11].

## Data Availability

The data are available on request from the author.
